# Methyltransferase K-D-K-E motif influences the intercellular transmission of Newcastle disease virus

**DOI:** 10.1080/21505594.2023.2186336

**Published:** 2023-03-15

**Authors:** Xiao Li, Ye Zhao, Qing-Yuan Teng, Xue-Hui Zhang, Jia Xue, Guo-Zhong Zhang

**Affiliations:** Key Laboratory of Animal Epidemiology of the Ministry of Agriculture, College of Veterinary Medicine, China Agricultural University, Beijing, China

**Keywords:** Newcastle disease virus, K-D-K-E motif, cell-to-cell, tunnelling nanotubes

## Abstract

We previously demonstrated that two methyltransferase motifs, K-D-K-E and G-G-D, affect the pathogenicity of Newcastle disease virus (NDV) by regulating mRNA translation and virus transmission. Here, we compared the infectious centre area produced by the NDV strain, rSG10, and methyltransferase motifs mutant rSG10 strains in DF-1 cells. The results show that intercellular transmission was attenuated by methyltransferase motif mutations. We further determined the ability of mutant viruses to spread in cell-free and cell-to-cell situations. Cell-free transmission of rSG10-K1756A was not reduced, indicating that cell-to-cell transmission of rSG10-K1756A was decreased. Using a donor and target system, we demonstrated that NDV can spread from cell-to-cell directly. Furthermore, by comparing the protein distribution area of three strains when treated with 2% agar overlay, we found that rSG10-K1756A was defective in cell-to-cell transmission. Tunnelling nanotubes (TNTs) are an important mode for cell-to-cell transmission. Treatment of cells with cytochalasin D (CytoD) or nocodazole to inhibit the formation of TNTs, reduced protein levels in all strains, but rSG10-K1756A was the least affected. These results indicate that mutation of the K-D-K-E motif is likely to restricted the spread of NDV via TNTs. Finally, we observed that matrix protein (M) and fusion protein (F) promoted the formation of cellular extensions, which may be involved in the cell-to-cell spread of NDV. Our research reveals a novel mechanism by which methyltransferase motifs affect the cell-to-cell spread of NDV and provides insight into dissemination of paramyxoviruses.

## Introduction

Non-segmented, negative-stranded (NNS) RNA viruses, such as vesicular stomatitis virus, Ebola virus, human metapneumovirus, and Newcastle disease virus (NDV), can cause serious diseases in humans and/or animals [1–4]. NDV belongs to the genus *Orthoavulavirus* in the family *Paramyxoviridae* and can infect most bird species [5]. Since the first discovery of Newcastle disease (ND) in Indonesia in 1926, it has spread around the world. NDV has caused several pandemics and remains a major threat to the present poultry industry [6–8].

The NDV genome encodes six structural proteins, of which large polymerase protein (L) is the largest and contains six functional domains. NNS RNA viruses contain two conserved methyltransferase motifs (G-G-G-D and K-D-K-E) in domain VI [9,10]. Methyltransferase motifs are also found in nsp14 of coronavirus and NS5 of flavivirus, indicating that methyltransferase motifs are present in a wide range of viruses [11–14]. The methyltransferase motifs play a critical role in methylation of the mRNA cap, which affects the replication and pathogenicity of viruses [15,16], while 2′-O methylation is essential for evasion of host immune responses [17–20]. We previously found mutation of two methyltransferase motifs, K-D-K-E and G-G-D, attenuates NDV pathogenicity by regulating mRNA translation and intercellular transmission [21]. Mutation of the K-D-K-E motif can increase cap-dependent translation to promote translation of mRNA. However, the mechanism by which methyltransferase motifs regulate intercellular spread remains unknown.

The intercellular spread of virus has significant impact on viral proliferation especially for low-dose infections. In the first cycle of infection, a small amount of virus invades a few cells. The virus then replicates in cells and spreads from infected cells to uninfected cells. The ability to replicate and to spread intercellularly determine the infection course of a virus. There are two main intercellular transmission mechanisms. One is the release of complete infectious virus particles from infected cells, which travel by liquid phase diffusion and are then adsorbed onto surfaces of other cells by binding to target cell surface receptors [22]. This spread mechanism of free viral particles is called cell-free spread. Another mechanism is through direct cell-to-cell transmission, such as via tunnelling nanotubes (TNT) or extracellular vesicles [23–25]. Virus particles transmitted by direct cell-to-cell spread do not contact the extracellular environment and can effectively evade the host’s immune response [26–28]. Many viruses can be transmitted from cell to cell, such as human metapneumovirus and influenza A viruses [29,30]. Virus-infected organelles can also transfer directly from cell-to-cell. Intercellular transfer of virus-infected mitochondria facilitated intercellular spread of porcine reproductive and respiratory syndrome virus and induced cell death [23].

As mentioned above, methyltransferase motifs, K-D-K-E and G-G-D, can influence the pathogenicity of NDV and the effect of the K-D-K-E motif was stronger than that of the G-G-D motif [21]. Mutation of the K-D-K-E motif promotes activation of cap dependent translation to benefit viral replication. Here, using rSG10-K1756A (this strain has a mutation in first amino acid of the K-D-K-E motif) and rSG10-G1780A (this strain has mutation in first amino acid of the G-G-D motif), we explored how methyltransferase motifs regulate the intercellular spread of NDV. Our research indicates a novel function of methyltransferase motifs and provides insight for the studies of methyltransferase motifs in other viruses.

## Materials and methods

### Cells and viruses

DF-1 cells (a chicken embryo fibroblast cell line), BSR-T7/5 cells (a baby hamster kidney cell line stably expressing T7 RNA polymerase) and Vero cells (an African green monkey kidney cell line) were cultured in Dulbecco’s modified Eagle’s medium (DMEM; Gibco, Grand Island, NY, USA) with 10% foetal bovine serum (FBS; Gibco) at 37°C in a 5% CO_2_ incubator (Thermo Forma, Marietta, OH, USA). The NDV virulent strains rSG10 (class II, genotype VII), the NDV recombinant strains rSG10-K1756A and rSG10-G1780A were recovered and preserved in our laboratory.

### Antibodies and drugs

A mouse monoclonal antibody against M protein and mouse polyclonal antibodies against NP protein and F protein were prepared in our laboratory. Anti-Flag, anti-β-actin, anti-α/β-tubulin, Alexa Fluor 488-conjugated anti-mouse IgG (H+L) and Alexa Fluor 555- conjugated anti-rabbit IgG (H+L) were purchased from Cell Signaling Technology (Beverly, MA, USA). The secondary antibodies against mouse or rabbit used for western blotting were purchased from Bioss Biotechnology (Beijing, China). 4′,6-diamidino-2-phenylindole (DAPI) was purchased from Sigma-Aldrich (St. Louis, MO, USA). CCK-8 cell proliferation and cytotoxicity assay kit were purchased from Solarbio Life Sciences (Beijing, China). CytoD and nocodazole were purchased from Absin Biotechnology (Shanghai, China). Phalloidin-TRITC Conjugate was purchased from AAT Bioquest (Sunnyvale, CA, USA).

### QRT-PCR

Total RNA was extracted using Premix Ex Taq reagents (TaKaRa, Dalian, China) from which cDNA was prepared. Quantitative real-time (qRT)-PCR was performed with previous primers as described earlier [31,32]. The comparative threshold cycle method was used to analyse the relative expression of RNA. All experiments were performed in triplicate. Expression of the NP gene was detected and normalized against that of GAPDH, and the value of rSG10 was set to 1.

### Indirect immunofluorescence assay (IFA) and confocal microscopy

Cell samples were harvested at indicated time points after infection and fixed with 4% paraformaldehyde. After infiltration with 0.25% Triton X-100 and blocking with 5% bovine serum albumin, the cells were incubated with indicated primary antibodies at 4℃ for 12 h and stained with Alexa Fluor 488-conjugated anti-mouse IgG (H+L) and/or Alexa Fluor 555- conjugated anti-rabbit IgG (H+L) (Cell Signaling Technology) at room temperature for 1 h. The nuclei were stained with DAPI (Sigma-Aldrich). The cells were washed five times with PBST (5 min/wash), then observed and photographed on a Nikon A1 fluorescence microscope (Nikon, Tokyo, Japan).

### Detection of adsorption, internalization and release ability

#### Adsorption

Cells were infected by each virus at different MOIs. Cells were incubated with virus at 4℃ for 1 h, then washed 10 times with precooled PBS. Relative NP RNA levels were determined by qRT-PCR. The housekeeping gene used to normalize the results was GAPDH. The 2^−∆∆CT^ value of rSG10 was defined as 1. The 2^−∆∆CT^ values of rSG10-K1756A and rSG10-G1780A were compared with the 2^−∆∆CT^ value of rSG10.

#### Internalization

Cells were infected by each virus at different MOIs and incubated at 4℃ for 1 h. Cells were then washed 10 times with precooled PBS. After adding the normal inoculum, cells were incubated at 37℃ for 1 h. Cells were washed 10 times with PBS and then treated with protease K (0.5 mg/mL) for 5 min to remove the virus particles adsorbed to the cell surface but not internalized. Relative levels of NP RNA were determined by qRT-PCR. The housekeeping gene used to normalize the results was GAPDH. The 2^−∆∆CT^ value of rSG10 was defined as 1. The 2^−∆∆CT^ values of rSG10-K1756A and rSG10-G1780A were compared with the 2^−∆∆CT^ value of rSG10. Internalization ability was defined by the ratio of internalization to adsorption.

#### Release

Cells were infected by each virus at different MOIs and the supernatant was collected at each time point to determine viral titres. The cells were washed three times with PBS and then the same volume of DMEM as the supernatant was added. After freezing and thawing three times, the viral titre was determined as the tissue culture infectious dose (TCID_50_). The release ability was defined as the ratio of the supernatant TCID_50_ to the lysed cell TCID_50_.

### Donor and target system

Vero cells were infected with rSG10-EGFP at an MOI of 0.01 for 36 hpi and then incubated with 7 μM cell tracker orange CRMA (Thermo Fisher Scientific, Waltham, MA, USA) for 30 min at 37℃. Cells were washed 5–10 times with PBS and collected after digestion with trypsin. These cells were then added to uninfected Vero target cells at a ratio of 1:1. Positive serum was added and cells were co-cultured for an additional 24 h. DMSO was added in control group instead of positive serum. Cells were then collected and fixed in 1% formaldehyde for flow cytometric analysis. The EGFP-only-positive cells were newly infected cells via direct cell-to-cell spread. Direct cell-to-cell spread was defined as the percentage of EGFP-only-positive target cells normalized to the percentage of double-positive donor cells.

### Western blotting

Vero cells were infected with rSG10, rSG10-K1756A, or rSG10-G1780A and harvested at indicated time points. Protein samples were prepared using the ProteinExt_®_ Mammalian Total Protein Extraction Kit (Transgen, Beijing, China), separated by 10% sodium dodecyl sulphate polyacrylamide gel electrophoresis (SDS-PAGE) and transferred to polyvinylidene difluoride membranes (Amersham Biosciences, Freiburg, Germany). Membrane was blocked with QuickBlock™ Western (Beyotime, Beijing, China) for 30 min and subsequently incubated with primary antibody at 4°C overnight. After washing three times with TBST, the membranes were incubated for 1 h at room temperature with horseradish peroxidase (HRP)-conjugated secondary antibodies (Bioss Biotechnology; 1:10,000 dilution). Protein bands were then detected using ECL western blot detection reagents (CWBIO, Beijing, China).

### Construction of expression plasmids

A full-length infectious clone of rSG10 was constructed previously [33] and was used to generate cDNAs encoding NP, P, M, F, HN and L proteins. The cDNAs were cloned into pRK5-flag to generate pRK5-flag-NP, pRK5-flag-P, pRK5-flag-M, pRK5-flag-F, pRK5-flag-HN and pRK5-flag-L.

### Statistical analyses

All data were analysed with GraphPad Prism software version 6.0 (GraphPad Software Inc., San Diego, CA, USA). All values are the mean ± standard deviation (SD) of three independent experiments. Paired t-test, one-way and two-way ANOVAs were used to evaluate the significance of differences.

## Results

### Mutation of the methyltransferase motifs restrict the intercellular spread of NDV

**Figure 1.**
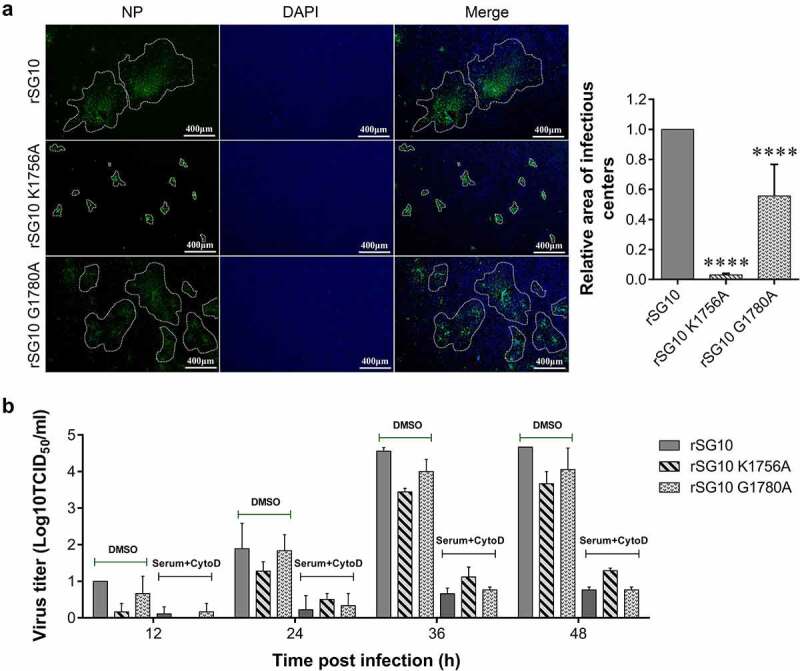
**Methyltransferase motifs restrict the intercellular spread of NDV**. (a) Difference of infectious centre area induced by rSG10 and mutant viruses. DF-1 cells were infected by rSG10 and mutant viruses. The relative infectious centre area of rSG10-K1756A and rSG10-G1780A were compared with that of rSG10. *P* values were calculated with a two-way ANOVA; *n* = 50; ****, *P* < 0.0001. (b) Assessment of the mainly intercellular spread mechanism of NDV. Vero cells were infected with each strain. Viral titres were quantified as the TCID_50_. The NDV positive serum dilution was 1:2. The experiment was performed in triplicate.

Mutations in methyltransferase motifs can affect the intercellular transmission of NDV in DF-1 cells [21]. To further investigate methyltransferase motifs, we examined the infectious centre areas formed by infection of DF-1 cells by each virus. The area of nucleocapsid protein (NP) distribution was enclosed with a white dotted line and the area was calculated by Image J. The largest infectious centre area was formed after rSG10 infection, which was approximately 33 times that of rSG10-K1756A and 2 times that of rSG10-G1780A (Figure 1a). These results indicated that the two methyltransferase motifs regulate the transmission of NDV to varying degrees *in vitro*, and that the effect of rSG10-K1756A was stronger than that of rSG10-G1780A when infected at 0.01 multiplicity of infection (MOI).

The intercellular transmission of viruses mainly depends on cell-free spread and cell-to-cell spread. To verify this for NDV, NDV positive serum was used to block the cell-free virions and cytochalasin D (CytoD) was used to block the formation of cell-to-cell connection by inhibiting the polymerization of F-actin. The viral titres were significantly reduced when treated with both serum and CytoD compared with DMSO treatment at different time points, especially at 36 and 48 hours post infection (hpi). This indicated that other NDV spread mechanisms may exist, but that cell-free and cell-to-cell transmission was dominant (Figure 1b).

### Decreased rSG10-K1756A transmission is not associated with cell-free spread

**Figure 2.**
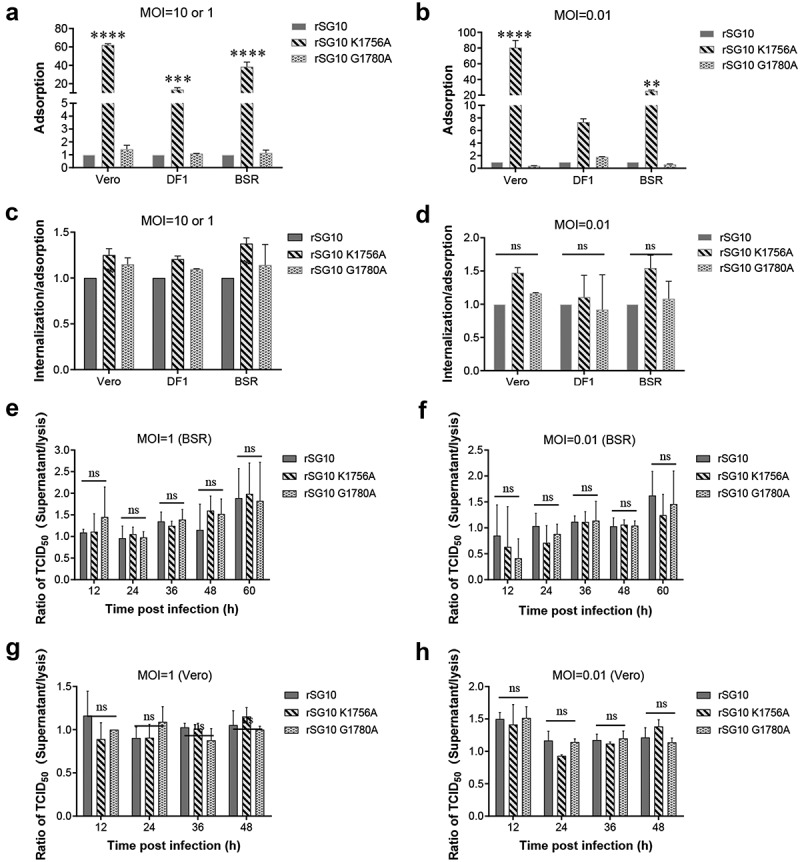
**Detection of the adsorption, internalization and release ability of rSG10 and mutant viruses**. (a – b) DF-1 cells were infected by each virus at an MOI of 10 or 0.01, BSR-T7/5 and Vero cells were infected at an MOI of 1 or 0.01 (viral titres in BSR-T7/5 cells and vero cells were lower than those in DF-1 cells and an MOI of 1 is the highest dose that can be achieved). The adsorption levels were detected as described in the materials and methods. (c – d) DF-1 cells were infected by each virus at an MOI of 10 or 0.01, BSR-T7/5 and Vero cells were infected at an MOI of 1 or 0.01. Internalization was detected as described in the materials and methods. (e – h) Cells were infected by each virus at different MOIs. The release levels were detected as described in the materials and methods. The Y-axis was defined as the ratio of the supernatant TCID_50_ to the lysed cell TCID_50_. *P* values were calculated with a two-way ANOVA; *n* = 3; ns, not significant; *, *P* < 0.05; **, *P* < 0.01; ***, *P* < 0.001; ****, *P* < 0.0001. All experiments were performed in triplicate.

To examine which mode of intercellular spread is affected by methyltransferase motifs, cell-free spread and cell-to-cell spread were separately detected. First, we detected the effect of methyltransferase motifs on cell-free spread. The cell-free process of virus transmission involves several infection steps, including adsorption, internalization, and release. We examined the adsorption and internalization of rSG10, rSG10-K1756A and rSG10-G1780A. The adsorption capacity was highest for rSG10-K1756A irrespective of the infection dose, high (10 or 1 MOI) or low (0.01 MOI), in different cell types (Figure 2a and b). In addition, the ratio of internalization to adsorption was used to evaluate the internalization ability and the results showed internalization ability of rSG10-K1756A was slightly higher or equal to that of rSG10 and rSG10-G1780A (Figure 2c and d). These results indicated that the entry of mutant strains into cells was not inhibited. We then evaluated differences in the release ability of mutant strains; however, there were no statistical differences among the three strains when infected at different MOIs (Figure 2e–h).

**Figure 3.**
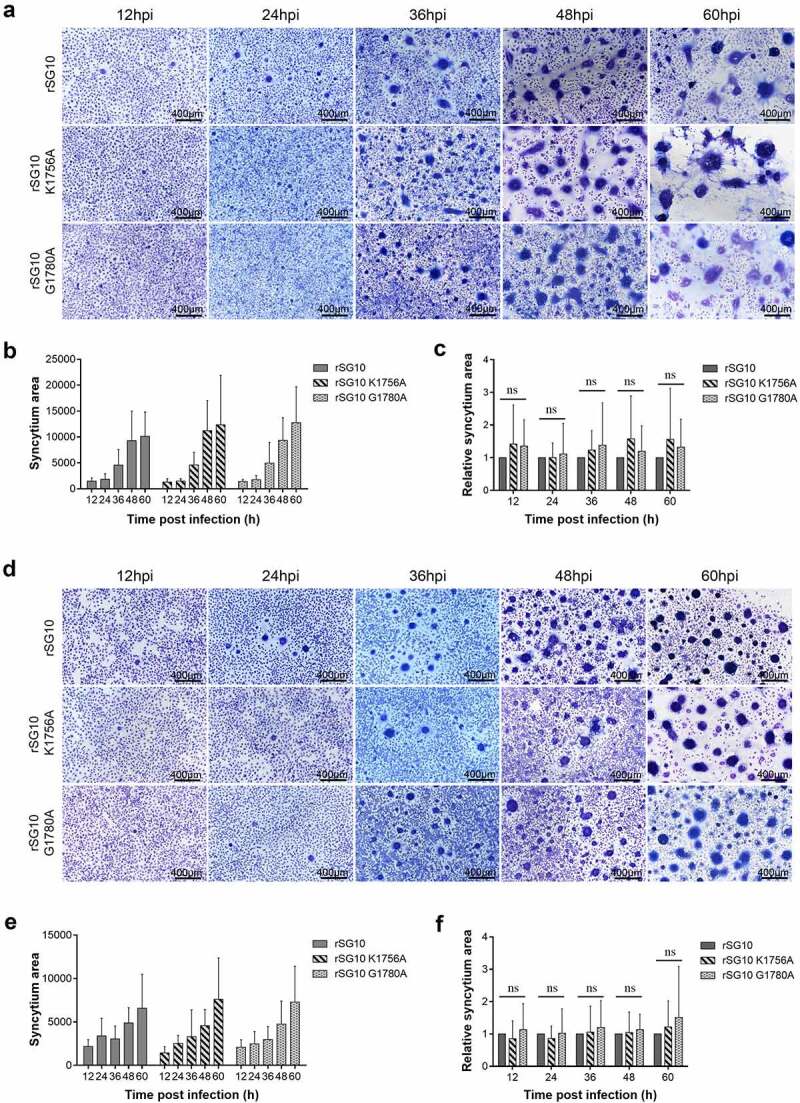
**Detection of the fusion activity of rSG10 and mutant viruses**. (a) Vero cells were infected by each virus at an MOI of 0.01. The cells were fixed with absolute methanol and stained with Giemsa solution. (b) Syncytium areas in (a) were analysed at different time points using Image J software, *n* = 100. (c) the syncytium area of rSG10 was defined as 1 in (a). The syncytium areas of rSG10-K1756A and rSG10-G1780A were compared with that of rSG10. (d) BSR-T7/5 cells were infected by each virus at an MOI of 0.01. The cells were fixed with absolute methanol and stained with Giemsa solution. (e) Syncytium areas in (d) were analysed at different time points using Image J software, *n* = 100. (f) the syncytium area of rSG10 was defined as 1 in (d). The syncytium areas of rSG10-K1756A and rSG10-G1780A were compared with that of rSG10. *P* values were calculated with a two-way ANOVA; *n* = 100; ns, not significant.

The membrane fusion activity of coronavirus S protein plays an important role in influencing its cell-to-cell transmission [34]. NDV infection can also mediate membrane fusion of host cells to form syncytia. Here, we explored changes in membrane fusion ability after methyltransferase motif mutation, and assessed the relationship between membrane fusion ability and intercellular transmission of mutant strains. With prolongation of infection time, the syncytium area gradually increased for each virus. However, there were no significant differences in relative syncytium area among these strains at different time points, showing that fusion activity was similar for each strain in Vero cells (Figure 3a and b). A similar phenomenon was observed in BSR-T7/5 cells (Figure 3d–f). These results demonstrated that cell-free spread did not contribute to the decreased transmission ability of rSG10-K1756A.

### NDV can spread directly from cell-to-cell

**Figure 4.**
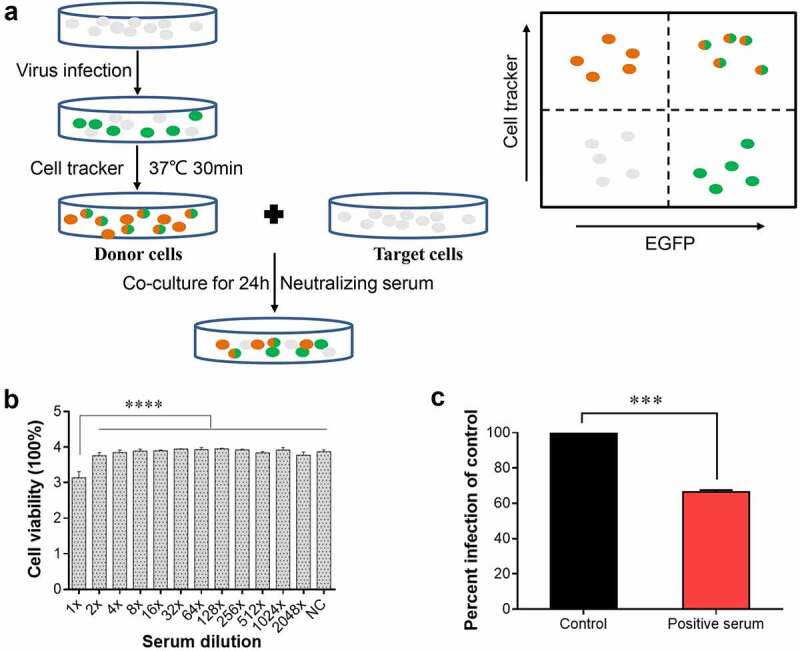
**NDV can spread directly from cell-to-cell**. (a) Schematic of the donor and target system. (b) Cell viability after treatment with positive serum at different dilutions. X-axis indicates serum dilutions. The NC group was set as a control, to which positive serum was not added. *P* values were calculated with an ordinary one-way ANOVA; *n* = 3; ****, *P* < 0.0001. (c) Percent infection was defined as the percentage of EGFP-only-positive target cells normalized to the percentage of double-positive donor cells. The NDV positive serum dilution was 1:2. The percent infection of the control was defined as 100%. *P* values were calculated with a paired t-test; *n* = 3; ***, *P* < 0.001.

After excluding an effect of rSG10-K1756A on cell-free spread, we focused on the effect of methyltransferase motifs on cell-to-cell spread of NDV. To verify that NDV can spread directly from cell-to-cell, we developed a donor and target system. EGFP-labelled cells represented the newly infected target cells. Double-labelled EGFP-positive (green) and cell tracker-stained (red) cells represented the originally infected donor cells (Figure 4a).

To test direct cell-to-cell spread of NDV, cells were co-cultured in the presence of neutralizing serum. To detect the neutralizing activity of serum, Vero cells were infected by rSG10 at an MOI of 1 or 0.01 and then incubated with gradient dilution serum for 48 h. Supernatants were collected to detect the viral titres and infect Vero cells for analysis the expression of viral protein. No titre or viral protein expression was detected indicating that the cell-free virions were completely neutralized. The serum diluted no more than 32 times inhibited infection by cell-free NDV particles, while serum diluted at least two times had no effect on cell viability (Figure 4b). Hence, the diluted NDV positive serum of 1:2 was used to neutralize cell-free virion particles. When positive serum was used in co-culture, 66% of intercellular transmission events still occurred compared with the DMSO treatment group. These results showed that NDV can spread directly from cell-to-cell in the presence of positive serum (Figure 4c), although the spread efficiency was reduced compared with the control. These results indicated that NDV can spread directly from cell-to-cell.

### Mutation in the K-D-K-E motif restricts cell-to-cell spread of NDV

**Figure 5.**
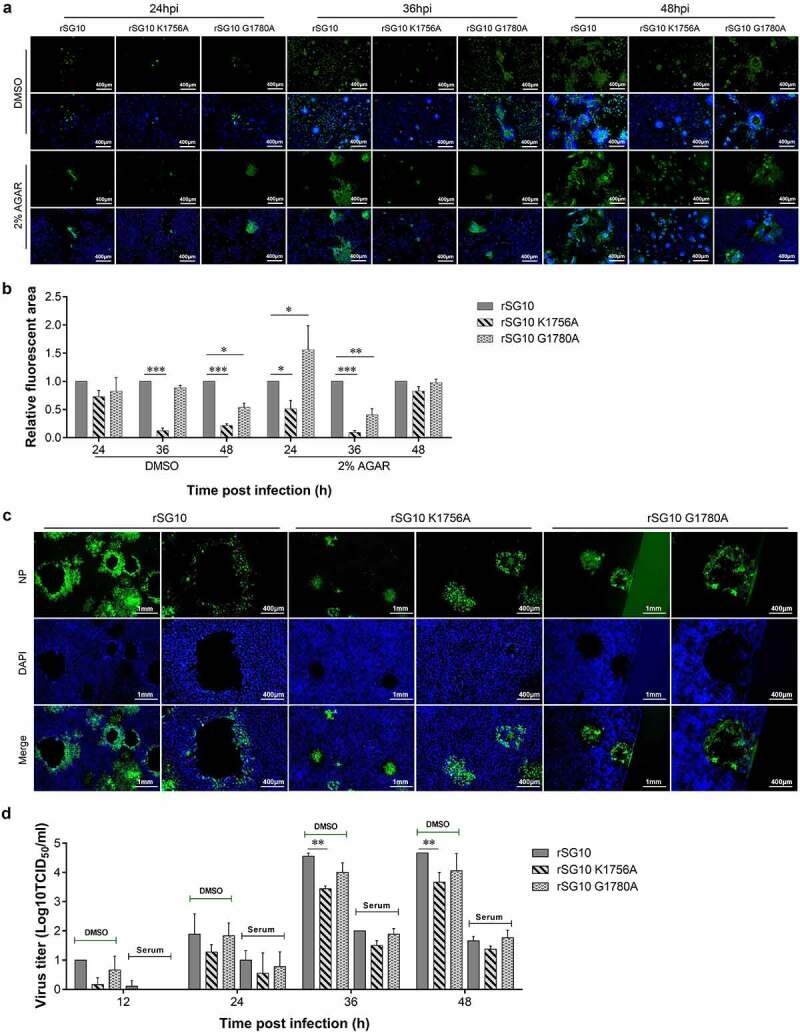
**Mutation in the K-D-K-E motif restricts cell-to-cell spread of NDV**. (a) NP distribution of each virus in the absence of 2% agar overlay. BSR-T7/5 cells were infected with each virus at an MOI of 0.01. After 1 h of adsorption, the inoculum was removed and replaced with an overlay medium containing 4% FBS and 2% agar in agar treatment group. Cells were left at room temperature for 30 min until the agar solidified and plates were then put in the incubator for the specified time. The overlay was then picked out and cells were analysed by IFA at 36 hpi. NP protein was detected with anti-NP antibody (Green) and DAPI was used to stain nuclei. (b) Fluorescent areas in (a) were measured using Image J software. The fluorescent area of rSG10 was defined as 1. The relative fluorescent area of rSG10-K1756A and rSG10-G1780A were compared with that of rSG10. (c) BSR-T7/5 cells were infected with each virus at an MOI of 0.001 in 6-well plates. After adsorption for 1 h, the inoculum was removed and replaced with an overlay medium containing 4% FBS and 2% agar. After infection for 5 days, the cells were washed with PBS and fixed with 4% formaldehyde for 6 h after picking out the overlay. IFAs were then performed to detect the distribution of NP protein in plaques. (d) Detection of cell-to-cell spread using positive serum. Vero cells were infected with each strain at an MOI of 0.01. After incubation with viruses for 1 h, cells were treated with NDV positive serum with a 1:2 dilution. The DMSO treatment group was prepared as a control. The supernatants of infected Vero cells were collected at the indicated time points. Viral titres were quantified as the TCID_50_. *P* values were calculated with a two-way ANOVA; *n* = 3; *, *P* < 0.05; **, *P* < 0.01; ***, *P* < 0.001. All experiments were performed in triplicate.

To initially examine whether methyltransferase motifs influence cell-to-cell spread in NDV infection, we tested virus spread, in the presence or absence (DMSO) of a 2% AGAR overlay media to prevent diffusion of cell-free virus particles when infected at an MOI of 0.01. In the DMSO treatment group, protein distribution of rSG10-K1756A was less than that of other viruses at different time points, which indicated the intercellular spread of rSG10-K1756A was weaker than that of other viruses. Meanwhile, the protein distribution of rSG10-K1756A was also less than that of rSG10 and rSG10-G1780A when overlayed with 2% agar to block all spread mechanisms that require a liquid medium. These results showed that protein distribution of rSG10-K1756A was restricted after cell-free spread was interrupted (Figure 5a and b).

NP protein distribution of the virus was detected before cells were shed (Figure 5a). With prolongation of culture time, a single virion spread outwards from the infection centre to infect adjacent cells and induce cell death and shedding to form a plaque. To confirm that formation of the plaque was directly caused by virus infection, agar was removed for immunostaining. NP protein was distributed throughout the plaque, even at the edge, except for a blank area left by cell loss (Figure 5c).

Finally, we further compared the ability of each virus for cell-to-cell spread by blocking cell-free virions with positive serum. The viral titre of each virus was reduced approximately 100-fold after treatment with positive serum for 36 and 48 h. The viral titre of rSG10-K1756A was lower than those of rSG10 and rSG10-G1780A in the DMSO treatment group. After serum treatment, the virus titre of rSG10-G1756A was lower than that of rSG10, although this difference was not statistically significant (Figure 5d). These results indicated that direct intercellular transmission of NDV was decreased by K-D-K-E motif mutation.

### NP protein is located in TNTs

**Figure 6.**
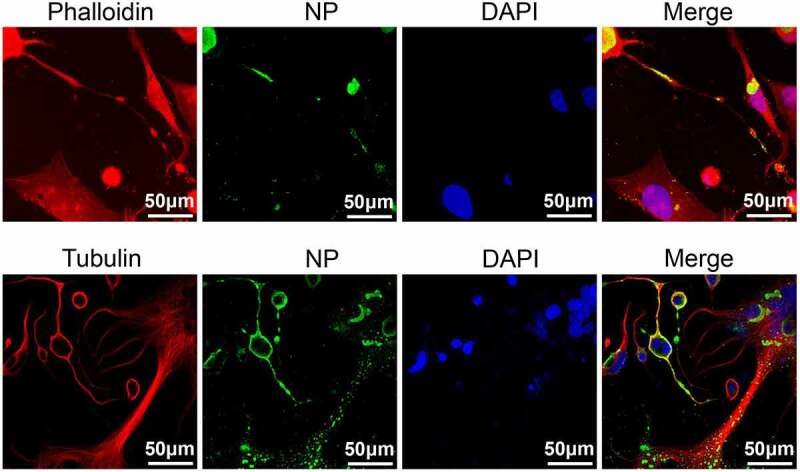
NP protein located in TNTs. Vero cells were infected by rSG10 at an MOI of 0.01 for 36 h and cells were analysed by IFA. DAPI was used to stain the nuclei, an anti-NP antibody was used to stain the NP protein of NDV, phalloidin was used to label F-actin and an anti-tubulin antibody was used to stain α/β-tubulin protein.

There are several direct cell-to-cell transmission mechanisms involving TNTs or extracellular vesicles. We excluded any spread mechanism that requires a liquid medium by overlaying with 2% agar (Figure 5). Viruses can spread via TNTs, which does not require a liquid medium. Firstly, we detected whether NDV can localize to TNTs. TNTs are formed by the outward extension of the cell membrane and is made up of cytoskeletal proteins such as F-actin and tubulin. Phalloidin is a polypeptide isolated from *Amanita phalloides*, a poisonous mushroom. It binds tightly to and stabilizes F-actin. Our results showed that intercellular TNT junctions can form between Vero cells after rSG10 infection. The NP protein of rSG10 was distributed on TNTs composed of both F-actin and tubulin (Figure 6). This indicated that TNTs formation upon NDV infection may help in cell-to-cell spread of virus.

### Mutated K-D-K-E motif inhibits NDV to use TNTs for cell-to-cell spread

**Figure 7.**
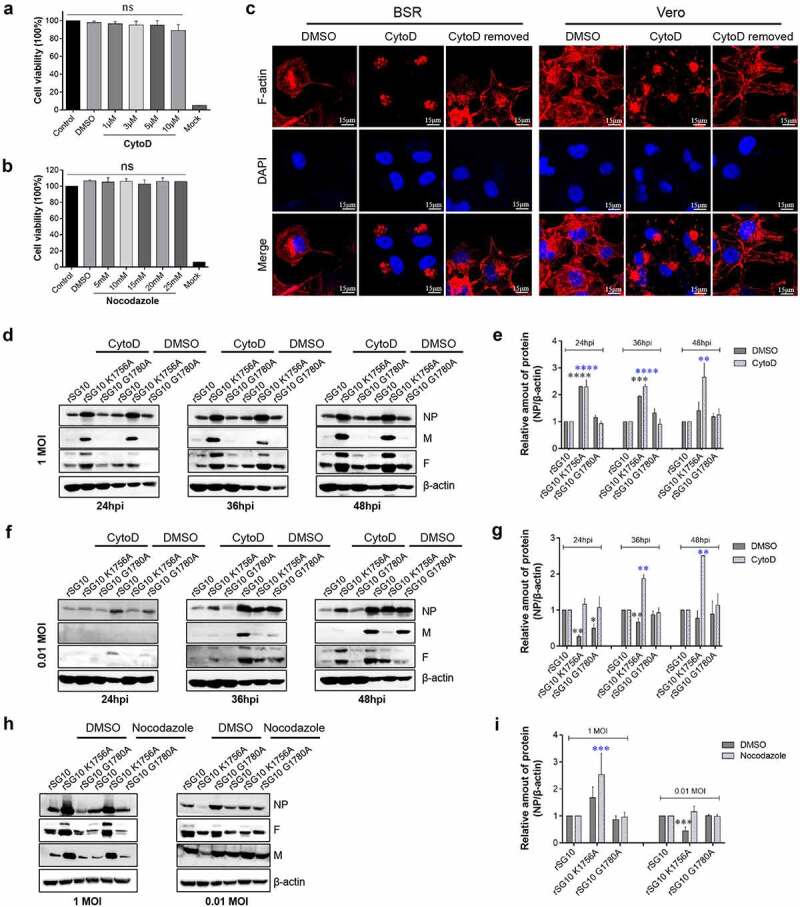
**Effects of methyltransferase motifs on the cell-to-cell spread of NDV via TNTs by western blotting**. (a – b) CCK-8 kit assessment of the effect of CytoD and nocodazole on cell viability. The untreated group (control) and DMSO treatment group were prepared as positive controls. A mock group that did not receive the CCK-8 kit reagent was prepared as a negative control. The cell viability of the Control group was defined as 100%. *P* values were calculated with an ordinary one-way ANOVA; n = 3; ns, not significant. (c) Morphology of Vero and BSR-T7/5 cells treated with 3 μM CytoD. Cells were treated with 3 μM CytoD for 12 h and then analysed by IFA. For the CytoD removed group, after treatment with cytoD for 12 h, the supernatant was removed and cells were washed three times. Then DMEM with 2% FBS was added and cells incubated for 3 h. (d) Differences in viral protein levels after infection at 1 MOI and CytoD treatment were detected by western blotting. Vero cells were infected by each virus and treated with 3 μM CytoD. At indicated time point, cells were collected and analysed by western blotting. (e) the relative greyscale value of the protein band in (d) was calculated by Image J software. The relative greyscale value of the rSG10 protein band at each time point was defined as 1. The relative greyscale values of the rSG10-K1756A and rSG10-G1780A protein bands were compared with those of the rSG10 protein bands at the corresponding time point in the DMSO/CytoD group. In the DMSO-treated group, *P* values are labelled by black *. In the CytoD-treated group, *P* values are labelled blue *. *P* values were calculated with a two -way ANOVA; n = 3; **, P < 0.01; ***, P < 0.001; ****, P < 0.0001. (f) Differences in the levels of viral proteins after infection and CytoD treatment were detected by western blotting. Vero cells were infected by each virus at 0.01 MOI and treated with 3 μM cytoD. At indicated time points, cells were collected and analysed by western blotting. (g) the relative greyscale value of the protein band in (f). *P* values were calculated with a two -way ANOVA; n = 3; *, P < 0.05; **, P < 0.01. (h) Differences in the levels of viral proteins after treatment with nocodazole were detected by western blotting. Vero cells were infected by each virus and treated with 15 mM nocodazole. At 48 hpi, cells were collected and analysed by western blotting. (i) the relative greyscale value of the protein band in (h). *P* values were calculated with a two-way ANOVA; n = 3; ***, P < 0.001. All experiments were carried out in triplicate.

To detect the effect of methyltransferase motifs on cell-to-cell spread of NDV via TNTs, inhibitors were used to block the formation of TNTs. CytoD is a potent F-actin polymerization inhibitor that stimulates F-actin depolymerization. Nocodazole is a rapidly reversible microtubule inhibitor. Nocodazole binds to β-tubulin and disrupts microtubule assembly/disassembly dynamics. Treatment with CytoD at 1, 3, 5 or 10 μM did not affect cell activity, but with more than 1 μM, the inhibitory effect was poor and some cells lost their spherical morphology (Figure 7a). Therefore, 3 μM was selected as the effective concentration. Treatment with 5 to 25 mM nocodazole did not significantly affect cell activity; therefore, as used in a previous study, 15 mM was selected as its effective concentration (Figure 7b). CytoD treatment caused F-actin to clump around the nucleus in BSR-T7/5 cells and Vero cells. After DMSO treatment, F-actin was distributed in the perinuclear and cell membrane. When the inoculum containing CytoD was removed and replaced with normal inoculum, the distribution of F-actin was consistent with that before adding CytoD, indicating that the inhibition of F-actin by CytoD was reversible (Figure 7c).

To evaluate the effect of methyltransferase motifs on the cell-to-cell transmission of NDV via TNTs, Vero cells were inoculated with each strain at different MOIs and incubated in inoculum supplemented with 3 μM CytoD. Western blotting was used to detect the effect of TNT formation inhibitors on the expression of viral proteins. At 24, 36 and 48 h after infection at an MOI of 1, the protein level trend in CytoD treatment and DMSO control groups was consistent. The protein levels of NP, M and F in rSG10-K1756A were higher than those in rSG10 and rSG10-G1780A because of the high replication ability of rSG10-K1756A, which is consistent with our previous study. (Figure 7d and e). This result confirmed our assumption that for infection at high dose, viral proliferation mainly depended on replication ability rather than intercellular spread ability. However, when the infection dose was 0.01 MOI, the protein level of rSG10-K1756A in the DMSO treatment group was lower than those of rSG10 and rSG10-G1780A at different time points, while the protein level of rSG10-K1756A after treatment with CytoD was higher than that of the other two strains. These results indicated that CytoD inhibited the polymerization of F-actin, which reduced the cell-to-cell transmission of the three strains. However, rSG10-K1756A was less affected than rSG10 and rSG10-G1780A. Since the replication capacity was stronger than that of rSG10 and rSG10-G1780A, the protein expression level of rSG10-K1756A was higher than that of other strains when infected at 0.01 MOI after the cell-to-cell transmission was inhibited, which indicated that K1756A mutation restrained the cell-to-cell transmission of NDV (Figure 7f and g). The protein expression trend after nocodazole treatment was consistent with that of CytoD. The protein level of rSG10-K1756A was higher than that of rSG10 and rSG10-G1780A at 48 hpi when infected at an MOI of 1, irrespective of treatment with DMSO or nocodazole. However, when the infection dose was 0.01 MOI, the protein level of rSG10-K1756A was lower than that of rSG10 and rSG10-G1780A after treatment with DMSO. After nocodazole treatment, the protein level of rSG10-K1756A was slightly higher than that of rSG10 and rSG10-G1780A (Figure 7h and i). These results indicated that after nocodazole inhibited the polymerization of microtubules, the protein expression was reduced for all three viruses, but that rSG10-K1756A was less affected.

**Figure 8.**
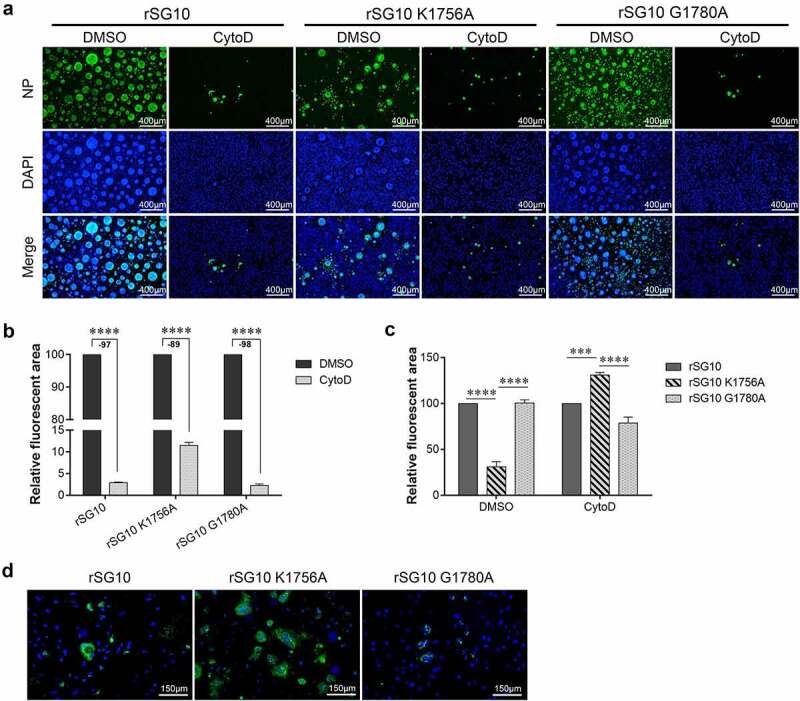
**Effect of methyltransferase motifs on the cell-to-cell spread of NDV via TNTs by IFA**. (a) IFA analysis of protein distribution after CytoD treatment. Vero cells were infected by each virus and treated with CytoD for 36 h. Afterwards, cells were fixed and analysed by IFA. Anti-NP antibodies were used to detect NP protein and DAPI was used to stain nuclei. (b) the fluorescent area was analysed by Image J software. The fluorescent area of the DMSO group was defined as 100%. Minus numbers indicate decreased fluorescent area percentage in the CytoD group compared with the DMSO group. *P* values were calculated with a two-way ANOVA; n = 3; ****, P < 0.0001. (c) the fluorescent area of rSG10 was defined as 100%. *P* values were calculated with a two-way ANOVA; n = 3; ***, P < 0.001; ****, P < 0.0001. (d) Analysis of protein distribution after nocodazole treatment. Vero cells were infected by each virus and treated with nocodazole for 36 h. Afterwards, cells were fixed and analysed by IFA. Anti-NP antibodies were used to detect NP protein and DAPI was used to stain nuclei. All experiments were performed in triplicate.

We then detected the effect of cytoD on the protein distribution of each virus when infected at 0.01 MOI by IFA. Immunostaining was performed to observe the distribution of NP protein. Compared with the DMSO treatment group, the level of NP protein of each strain treated with CytoD was decreased at 36 hpi (Figure 8a). After CytoD treatment, the area of green fluorescence induced by rSG10 infection decreased by 97%, that of rSG10-G1780A decreased by 98%, and that of rSG10-K1756A decreased by 89% (Figure 8b). After DMSO treatment, the level of rSG10-K1756A NP protein was significantly lower than that of rSG10 and rSG10-G1780A, while the green fluorescence area of rSG10-K1756A after CytoD treatment was significantly larger than that of rSG10 and rSG10-G1780A (Figure 8c). The same results were also observed after treatment with 15 mM nocodazole. The green fluorescence area induced by rSG10-K1756A was larger than that induced by rSG10 and rSG10-G1780A (Figure 8d). These results showed that the sensitivity of rSG10-K1756A to CytoD and nocodazole was low. Therefore, although protein expression of rSG10 and mutant strains was reduced, rSG10-K1756A was least affected.

### Viral proteins induce formation of the cellular extension

**Figure 9.**
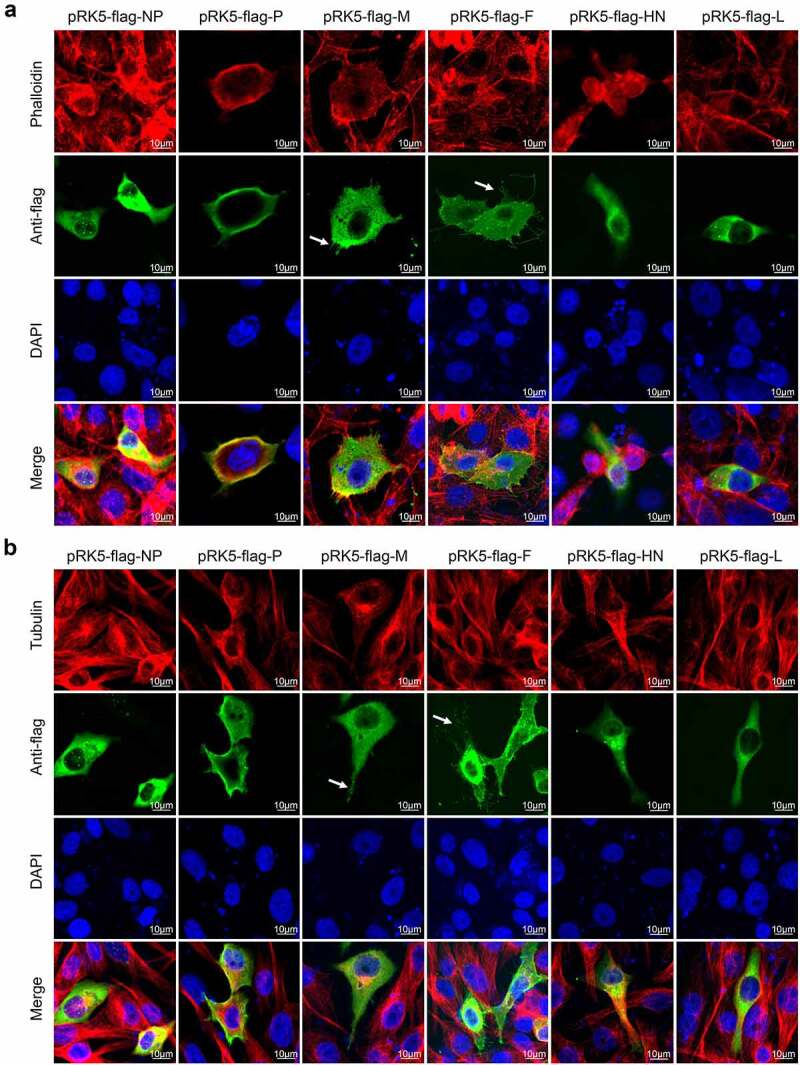
NDV M and F proteins induce formation of cellular extensions.(a) Vero cells were transfected with pRK5-flag-NP, pRK5-flag-P, pRK5-flag-M, pRK5-flag-F, pRK5-flag-HN or pRK5-flag-L. At 36 hpt, cells were processed for IFA. Viral proteins were detected with anti-flag antibodies, F-actin was detected using phalloidin and DAPI was used to stain nuclei. (b) Vero cells were transfected with pRK5-flag-NP, pRK5-flag-P, pRK5-flag-M, pRK5-flag-F, pRK5-flag-HN or pRK5-flag-L. At 36 hpt, cells were processed for IFA. Viral proteins were detected with anti-flag antibodies, tubulin was detected with anti-α/β-tubulin antibodies and DAPI was used to stain nuclei.

TNTs are formed by the cell membrane. After low-dose NDV infection, the cell membrane extends outwards to form the TNT through which the virus can then spread. To clarify which viral proteins are involved in TNT formation, Vero cells were transfected with pRK5-flag-NP, pRK5-flag-P, pRK5-flag-M, pRK5-flag-F, pRK5-flag-HN or pRK5-flag-L to express NP, phosphoprotein (P), M, F, haemagglutinin-neuraminidase protein (HN) and L protein. At 36 hours post transfection (hpt), IFAs were performed to observe cell morphology. Thin and long extensions consisting of F-actin and tubulin were observed around the cell after transfection with pRK5-flag-F. M, P and HN proteins also promoted the formation of cell extensions, although these extensions were fewer and short. The NP and L proteins had no obvious effect on the formation of cell extensions. These results indicate that viral proteins, especially F protein promotes the formation of externally extensions from the cell membrane (white arrows), which may be involved in cell-to-cell spread of NDV (Figure 9a and b).

## Discussion

The ability of a virus to replicate and transmit is a key factor affecting virus proliferation. We previously showed that for high-dose infection the virus titre of rSG10-K1756A was higher than that of rSG10, indicating that replication of NDV was enhanced by the K-D-K-E motif mutation. However, for low-dose infection, the virus titre of rSG10-K1756A was lower than that of rSG10. According to Poisson distribution, the virus titre was mainly determined by replication and transmission ability for low-dose infection [21]. Based on the enhanced replication capacity of high-dose rSG10-K1756A, we speculated that the K-D-K-E motif mutation reduces intercellular transmission of NDV, resulting in a lower viral titre than rSG10 after low-dose infection.

To verify whether the transmissibility of the mutant strain was changed, we analysed the infectious centre area and showed that methyltransferase motif mutations can reduce intercellular transmission of NDV to varying degrees. On the one hand, this finding confirms our prediction, and on the other hand, expands our understanding of methyltransferase motifs. The methyltransferase motifs are mainly responsible for methylation of the viral mRNA cap structure [35–37]. Methyltransferase motif mutations can reduce the methylation level of G-N-7 and/or 2′-O in the mRNA cap structure and enhance the ability of the virus to evade the innate immune response of the host [11,13,18,19,38]. Here, we provide novel insight into the function of methyltransferase motifs in the intercellular spread of viruses.

The ability of a virus to spread between cells is important for its ability to proliferate. Our results showed that the cell-free spread of the mutant strains was not lower than that of the parent strain after infection at low dose. This indicates that the reduced transmissibility of the mutant strain results from the reduced transmissibility via direct contact between cells. Many viruses can be transmitted from infected cells to uninfected cells by direct cell-to-cell transmission [39–41]. Human immunodeficiency virus can be transmitted by both cell-free and direct cell-to-cell transmission, and mathematical models show that direct cell-to-cell transmission may be 100–1000 times faster than cell-free transmission [42,43]. Measles morbillivirus can transmit directly from neuron to neuron in NT2N cells [44] and hepatitis C virus and SARS-CoV-2 can transmit directly from cell-to-cell in the presence of neutralizing antibodies [34,45,46]. In this study, using a donor and target system, we demonstrated that NDV can also transmit directly from cell-to-cell. On this basis, we compared differences in direct cell-to-cell transmission among NDV strains with methyltransferase motif mutations by blocking cell-free transmission with a 2% agar overlay or positive serum. The results showed that cell-to-cell spread was reduced by the K-D-K-E motif mutation.

There are many routes by which viruses achieve direct cell-to-cell transmission, such as extracellular vesicles or TNTs [24,25,40,47]. Although vesicle transport between cells is direct transmission, it still requires a liquid medium. In contrast, TNTs, formed by the outward extension of the cell membrane, do not require a liquid medium [48]. TNTs were first described as a new intercellular communication mechanism in 2004 [49]. They are between 50 nm and 200 nm in diameter, and they can connect cells and transfer materials, such as organelles, ions, proteins and miRNAs, between cells [22,23,29,50–54]. Viruses can also spread via TNTs and some virus infections induce the formation of TNTs [55–57]. TNTs are formed by outward extension of the cell membrane and mainly consist of F-actin and tubulin. To verify whether methyltransferase motif mutations can inhibit NDV transmission through TNTs, the levels of viral proteins were detected after treatment with CytoD (an F-actin polymerization inhibitor) of nocodazole (a tubulin stabilizer). The level of rSG10-K1756A protein in the DMSO treatment group was lower than that of the parent strain after low-dose infection, while the level of rSG10-K1756A protein was higher than that of the parent strain after treatment with either inhibitor. Because mutated K-D-K-E motif (rSG10-K1756A) inhibits NDV to use TNTs for cell-to-cell spread, the spread of rSG10-K1756A was less affected by CytoD and Nocodazole. These results indicated that the K-D-K-E motif mutation have an inhibitory action on direct cell-to-cell transmission of NDV through TNTs.

Viruses are able to manipulate the cytoskeleton by encoding viral proteins that bind directly to actin or actin binding proteins. Some viral proteins are directly involved in cell-to-cell spread. P protein of human metapneumovirus can co-localize with F-actin and induce the formation of cellular extensions [29]. The cell-to-cell spread of measles morbillivirus between human neurons is dependent on haemagglutinin and hyperfusogenic fusion protein [44], while glycoprotein J is associated with viral replication and cell-to-cell spread in Duck plague virus [58]. Surface glycoprotein of Borna disease virus mediates effective virus spread in permissive cell cultures and also in natural host cells [59]. Envelope C-terminal amino acid composition regulates the ratio of cell-free to cell-cell transmission for Foamy virus [60]. The US2 protein is involved in penetration and cell-to-cell transmission of duck enteritis virus *in vitro* [61]. M protein of measles morbillivirus and NDV binds to actin [30,62]. Our research found that viral proteins induce changes to the plasma membrane and promote the formation of cellular extensions in Vero cells. M protein is involved in the assembly and budding of virions and F protein is associated with the transport and release of virions [63–65]. These two proteins have the potential to regulate and induce cell membrane deformation. However, the mechanism by which methyltransferase motif mutations affect the transmission of virus via TNTs remains unclear. It is unclear whether this is a direct function of methyltransferase motifs. The intercellular spread of rSG10-G1780A was slightly reduced (Figure 1a), but the cell-free and TNT-mediated cell-to-cell spread of rSG10-G1780A was not reduced. Does the G-G-D motif regulate the intercellular spread of NDV via non-cell-free or non-cell-to-cell mechanisms? These questions require further study.

In summary, we discovered how methyltransferase motif mutations affect the pathogenicity of NDV. Mutation of the G-G-D motif had no remarkable effect on NDV replication, but its capacity for intercellular transmission was slightly lower than that of the parent strain, which would result in a delayed disease course and reduced mortality after infection. Methyltransferase motifs are present in many viruses, we believe that our findings provide an important basis for research into the pathogenicity of other viruses.

## Data Availability

The data that support the findings of this study are available from the corresponding author upon reasonable request (zhanggz@cau.edu.cn).
